# Overexpression of DYRK1A in down syndrome: Analysis of kinase activity

**DOI:** 10.1016/j.bbrep.2026.102722

**Published:** 2026-07-25

**Authors:** Wieslaw K. Dowjat, Yu-Wen Hwang

**Affiliations:** a Department of Developmental Neurobiology, New York State Institute for Basic Research in Developmental Disabilities, Staten Island, NY, USA; b Department of Molecular Biology, New York State Institute for Basic Research in Developmental Disabilities, Staten Island, NY, USA

**Keywords:** Rapid DYRK1A purification, ELISA DYRK1A activity, GCG inhibition, DYRK1A phosphorylation

## Abstract

DYRK1A (dual-specificity tyrosine phosphorylation regulated kinase 1A) is known to play critical roles in regulating numerous cellular functions like cell cycle, neuronal development, and immune homeostasis. Altered expression DYRK1A is implicated in Alzheimer's Disease and various features of Down Syndrome (DS). The DYRK1A gene is located on chromosome 21 and due to trisomy of this chromosome in DS, the level of DYRK1A is elevated in the gene-dosage dependent manner. Nevertheless, how an increase in DYRK1A protein level would affect its activity in DS remains to be determined. The question was examined by performing side by side comparison of protein levels and the activity of DYRK1A extracted from control and DS samples. We show that DYRK1A activity elevation in DS is in parallel to that of DYRK1A protein level regardless of the status of kinase phosphorylation. This suggests that DYRK1A catalytic activity in DS is mainly regulated by protein level.

## Highlights

• DYRK1A activity is elevated in DS subjects in a gene-dosage dependent manner.
• The elevation of DYRK1A activity is in parallel with that of DYRK1A protein level in DS.
• The status of kinase phosphorylation does not affect DYRK1A activity.
• Protein level appears to be the main element controlling the activity of cellular DYRK1A.

## 1 Introduction

DYRK1A (dual-specificity tyrosine phosphorylation regulated kinase 1A) is the human ortholog of the Drosophila minibrain gene (MNB), which plays a crucial role in Drosophila postembryonic neurogenesis [1]. The DYRK1A gene is located on chromosome 21q22.13 region [2], which contains a subset of genes implicated in DS [3]. It is assumed that phenotypic features of DS stem from trisomy-driven altered expression of these genes [3]. DYRK1A levels in DS individual is increased in a manner (∼1.5-fold) reflecting the gene-dosage effect [[4], [5], [6]]. Elevated level of DYRK1A apparently underlie various abnormalities such as cognitive deficits, early onset of Alzheimer Disease (AD), diabetes and other symptoms associated with DS [7].

DYRK1A functions as a serine/threonine kinase toward exogenous substrates [[8], [9], [10]]. DYRK1A is able to phosphorylate tau at Thr-212 and primes it for subsequent phosphorylation by GSK3β [11,12]. Hyperphosphorylation drives tau aggregation and subsequent neurofibrillary tangle formation, a hallmark of AD [13]. DYRK1A can also phosphorylate amyloid precursor protein another key player of AD, at Thr-688 and promote β-secretase cleavage, possibly leading to increased production of amyloidogenic peptides and deposition of amyloid plaques [14]. Taken together, elevated DYRK1A may be the basis for the accelerated AD pathogenesis in DS. DYRK1A over-expression may also contribute to the high rate of diabetes seen in DS [15]. NFAT (nuclear factor of activated T-cells) is a critical regulator for promoting pancreatic beta cell proliferation and insulin production/secretion [16]. Like its action on tau, DYRK1A also works as a NFAT-priming kinase by phosphorylating NFAT at its conserved serine-proline motifs to facilitate further phosphorylation by GSK3β and CK1 [17]. These modifications impede NFAT activity in pancreatic beta cells, which may link directly to diabetes [18].

It is not clear how an increase in DYRK1A protein level and possibly other factors in the context of DS would affect its activity. We attempt to address this issue directly by conducting side by side comparison of protein levels and kinase activity in control and DS samples obtained from cell lines and brain tissues. The elevation of DYRK 1A protein level and activity were found to be parallel in DS. Our findings support the view that cellular DYRK1A activity directly reflects its protein level in DS.

## 2 Materials and Methods

*Materials.* Ni-NTA agarose was obtained from QIAGEN (Hilden, Germany). Imidazole, (−)GCG, and BCIP/NTB were purchased from Sigma-Aldrich (St. Louis, MO, USA). Anti-DYRK1A mAb 8D9 and anti-phospho-dynamin 1 mAb 3D3 were described [19,20]. Anti-anti-phospho-tyrosine (pY) mAb PY-99 and anti-phospho-threonine (pT) mAb p-Thr-Pro-101 were obtained from Santa Cruz Biotechnology (Dallas, TX, USA) and Cell Signaling Technology (Danvers, MA, USA), respectively. Alkaline phosphatase (AP)-conjugated goat-anti mouse secondary antibody was purchased from Jackson ImmunoResearch (West Grove, PA, USA). Alkaline phosphatase substrate PNPP and Pierce™ phosphatase inhibitor mini tablets were products of Thermo-Fisher Scientific (Waltham, MA, USA). Fetal bovine serum was obtained from Cytiva (Logan, UT, USA). RPMI 1640 medium and antibiotics were purchased from Thermo-Fisher Scientific (Waltham, MA, USA).

*Lymphoblastoid Cell Lines (LCLs) and Brain Tissues*. Normal EBV immortalized LCLs, GM03657, GM07041, GM07048, and ΑG09387, were obtained from the Coriell Cell Repositories (Camden, NJ, USA). DS cell lines DS19, DS68, and DS405, were established in the Department of Human Genetics of the New York State Institute for Basic Research by transforming peripheral blood mononuclear cells obtained from DS individual with EBV similarly as described [21]. Suspensions of lymphocyte cultures were maintained in RPMI 1640 medium supplemented with 10% fetal bovine serum and a mixture of penicillin and streptomycin. Cells after harvest were washed twice in cold PBS and stored as pellet at −70°C until use. Frontal cortex of normal and DS subjects was obtained from the Institute for Basic Research Brain Bank. Brain tissue was kept frozen at −70°C until use.

*Preparation of Brain and Lymphocyte Lysates.* Frozen dissected frontal cortex tissue was mortar ground in liquid nitrogen. One gram of powdered tissue was lysed in 1 mL of cold lysis buffer (50 mM sodium phosphate, pH 8.0, 300 mM NaCl, 20 mM imidazole, 0.05% Tween 20) containing *Complete* protease inhibitors (Roche, Indianapolis, IN, USA). After short pulse sonication, lysates were clarified by microfuge centrifugation at 16,000 xg for 5 min. Preparation of lymphocytes lysates was essentially the same as brain tissue by lyzing frozen pellets containing 2 x 10^7^ cells in 1 mL of cold lysis buffer. Protein concentration of lysates was determined by bicinchoninic acid assay as instructed by manufacturer.

*DYRK1A Purification*. Lysates were partial purified by Ni-NTA agarose as follows. 0.5 mL of precleared lysate containing 1 mg of total protein was mixed with 50 μL of Ni-NTA beads suspension in 1.5 mL microcentrifuge tube and rotated at 4°C overnight. Agarose beads were washed three times with cold lysis buffer followed by elution with 60 μL of 250 mM imidazole buffer.

*DYRK1A Protein Level Measurement*. Levels of DYRK1A in crude lysates and Ni-NTA eluents were measured by Western blotting semi-quantitatively as described [4]. Briefly, samples to be analyzed (20 μg total protein) were separated in 8% SDS-PAGE and blotted onto PVDF membrane. After blocking, membrane was probed with antibody 8D9 (1:2000 dilution) overnight, with anti-actin antibody when necessary, followed by alkaline phosphatase-conjugated secondary antibody. The immunoreactive bands were visualized by color reaction with BCIP/NBT and their densitometric measurement was carried out with ID Scan EX3.1 (Scanalytic Corp, Rockville, MD, USA). All DYRK1A splicing variants were quantified collectively. In case of crude lysates, DYRK1A signals were normalized to actin while in Ni-NTA eluents, to total loaded protein.

*DYRK1A Activity Measurement*. DYRK1A activity in the presence of imidazole and 2 μΜ GCG was measured by an ELISA-based assay using recombinant truncated DYRK1A (HT-497) as described [22]. DYRK1A activity in Ni-NTA eluents was measured similarly. For each Ni-NTA eluent sample, phosphorylation reactions were conducted in two parallel sets (each with duplicates), one set with 10 μM GCG and the other without, in 100 μL of reaction mixture. The reaction was initiated by the addition of 5 μL Ni-NTA eluent and allowed to proceed at 30°C for 30 min. Ni-NTA eluents were used directly and the variations in protein concentration were corrected afterward during activity calculation. A set of reaction controls catalyzed by HT-497 (0.5 ng), with and without GGC in the presence of 12.5 mM imidazole, were performed alongside. Phosphorylated substrate was first probed with mAb 3D3 [20] followed by secondary antibody for subsequent signal detection. The relative DYRK1A activity in the sample was obtained as follows: (readout of reactions without GCG)-(readout with GCG)/(readout of control reaction). The ratio of DYRK1A activity (either DS/normal or without/with PI) was calculated by dividing the relative activity of the former by that of the latter followed by correcting for the protein concentrations in Ni-NTA eluents. Only data from parallel reactions run simultaneously on the same plate were used for the ratio calculation.

*Phospho-Amino Acids Determination*. The level of phospho-amino acids in Ni-NTA eluents was determined by ELISA as follows. Eluents (2 μg total protein), obtained from cell lines GM07041 and GM07048, to be tested were diluted in 100 μL PBS and used to coat wells of a microtiter plate overnight at 4°C. After blocking with 2% BSA in PBS for 1 h at room temperature, wells were either probed with PY-99 (1:1000 dilution) or with p-Thr-Pro-101 (1:500 dilution) for 1 h at room temperature followed with conjugated secondary antibody (1:2000 dilution of 1 mg/mL solution) for 1 h at room temperature. Eluents prepared with and without PI were analyzed in parallel on the same plate. The alkaline phosphatase readouts were corrected for the background control (no eluent coating) for each antibody and then used for calculating the ratio of phospho-amino acids with versus without PI. Only data from parallel reactions run simultaneously on the same plate were compared.

## 3 Results

**Fig. 1:**
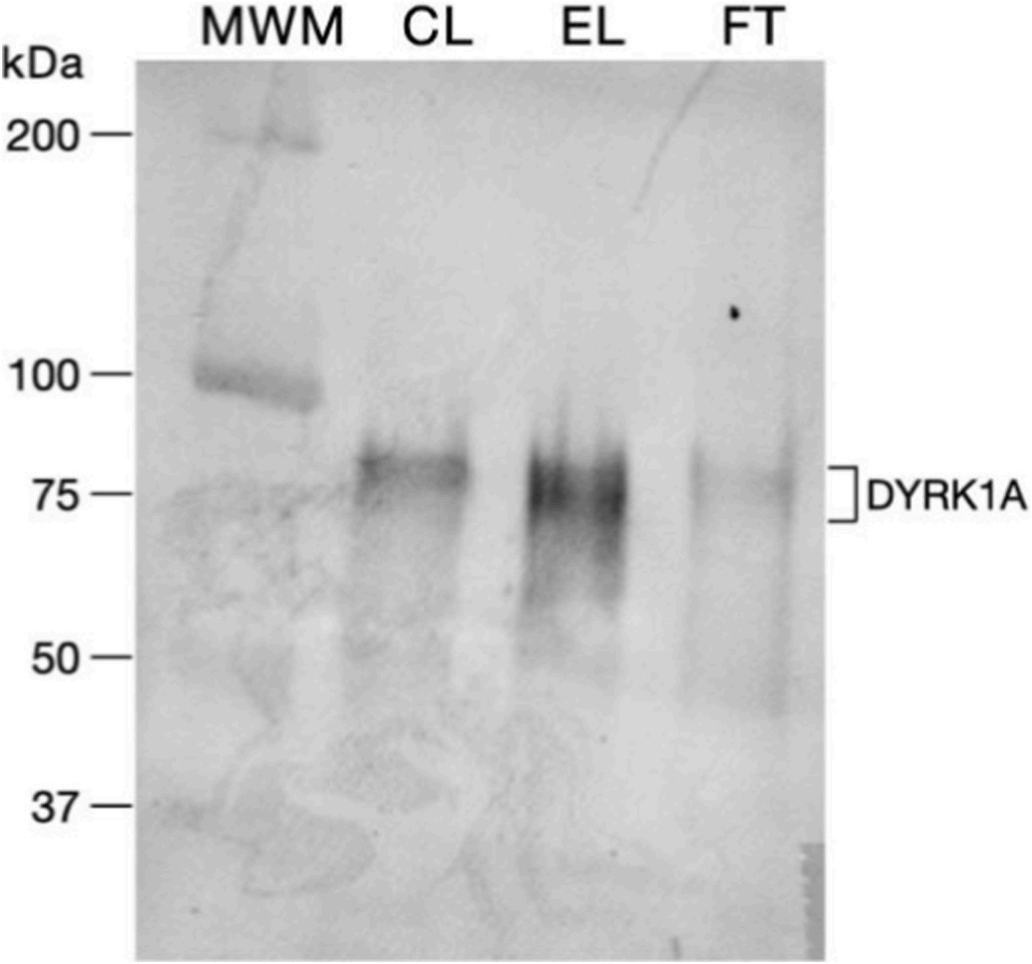
Purification of Cellular DYRK1A by Ni-NTA agarose beads. Brain lysates were prepared from tissues of normal individual and purified by Ni-NTA agarose as described in Materials and Methods. Aliquots from crude lysate (CL), imidazole eluents (EL) and flow through (FT) were stained in Western using anti-DYRK1A antibody 8D9. MWM, molecular weight markers.

*DYRK1A Assay.* DYRK1A is able to bind metal-chelating resins such as Ni-NTA because the polypeptide chain contains a stretch of 13 histidines [23]. Thus, DYRK1A in cell lysate can be rapidly enriched by Ni-NTA under native conditions (Fig. 1) before subjecting to activity measurement to reduce non-specific activity. Elution of DYRK1A from Ni-NTA was accomplished by supplementation with 250 mM imidazole (EL in Fig. 1). Under our purification conditions, greater than 90% of cellular DYRK1A are consistently pulled down from lysate in a single pass. Cellular DYRK1A produces multiple bands (Fig. 1) on a Western blot due to alternative splicing [24,25]. All of them appear to be enriched indiscriminately (Fig. 1). Nevertheless, splicing DYRK1A variants in Fig. 1 were not clearly distinguishable as those in Fig. 3, which were conducted using LCLs (Fig. 3A). This apparent degradation may be due to various uncontrollable factors of collecting post-mortem tissues.

To determine whether the eluents could be used directly in the established ELISA-based DYRK1A assay [22], we first examined whether the activity of DYRK1A is affected by imidazole. Imidazole with concentrations up to 25 mM appears to have little or no effect on DYRKA activity (Table 1). It suggests that Ni-NTA eluents could be used directly for the assay.

**Table 1:** *DYRK1A activity in the presence of imidazole and GCG*.

Imidazole	Relative DYRK1A Act. (%)	Relative DYRK1A Act. with 2 μM GCG (%)
0	100	7.92 ± 0.79
6.25	101.71 ± 4.43	8.13 ± 0.49
12.5	98.70 ± 1.96	7.74 ± 0.18
25	98.36 ± 2.32	8.55 ± 0.24

DYRK1A assay was conducted with indicated concentrations of imidazole and with or without GCG as described in Materials and Methods. The data represent DYRK1A activity at the indicated conditions relative to the activity without imidazole and GCG. The data were the mean of two independent experiments, each with triplicate samples.

We took another step attempting to further reduce the contribution of non-specific activity in the Ni-NTA fractions to the final readout. Gallocatechin gallate (GCG) is a potent and selective allosteric inhibitor of DYRK1A [26]. Our approach is to run the kinase assay in the presence of GCG at sufficiently high concentrations to inhibit nearly all DYRK1A activity and then to treat the portion that is inhibited by GCG as the actual DYRK1A activity. The feasibility of using GCG together with imidazole was analyzed in the ELISA-based assay using purified DYRK1A. As shown in Table 1, the activity of DYRK1A and the potency of GCG were not affected by imidazole at concentrations up to 25 mM. It should be noted that we performed the test assays only with 2 μM GCG, under which DYRK1A still displayed significant residual activity. If GCG's inhibition is in any way altered by imidazole, the consequence should be easy to observe.

With 10 μM GCG, purified DYRK1A is largely inhibited (97.35 ± 2.01%, n = 8); therefore, we employed this concentration of GCG for all subsequent assays. We routinely run the assays with 5 μL Ni-NTA eluent in 100 μL reaction mixture in two sets: one with GCG and one without. This results in a final concentration of 12.5 mM imidazole in the reaction, at which level imidazole is not expected to affect DYRK1A activity and GCG inhibition. Importantly, 5 μL Ni-NTA eluent was able to drive the phosphorylation reaction for easy detection. Under the specified conditions, about 80% phosphorylating activity of Ni-NTA eluents from both normal (78.97 ± 3.86%, n = 8) and Down syndrome (DS) (77.95 ± 5.36%, n = 8) cell lines was repressed by GCG compared to a no drug added normal and DS brain tissues.

**Fig. 2:**
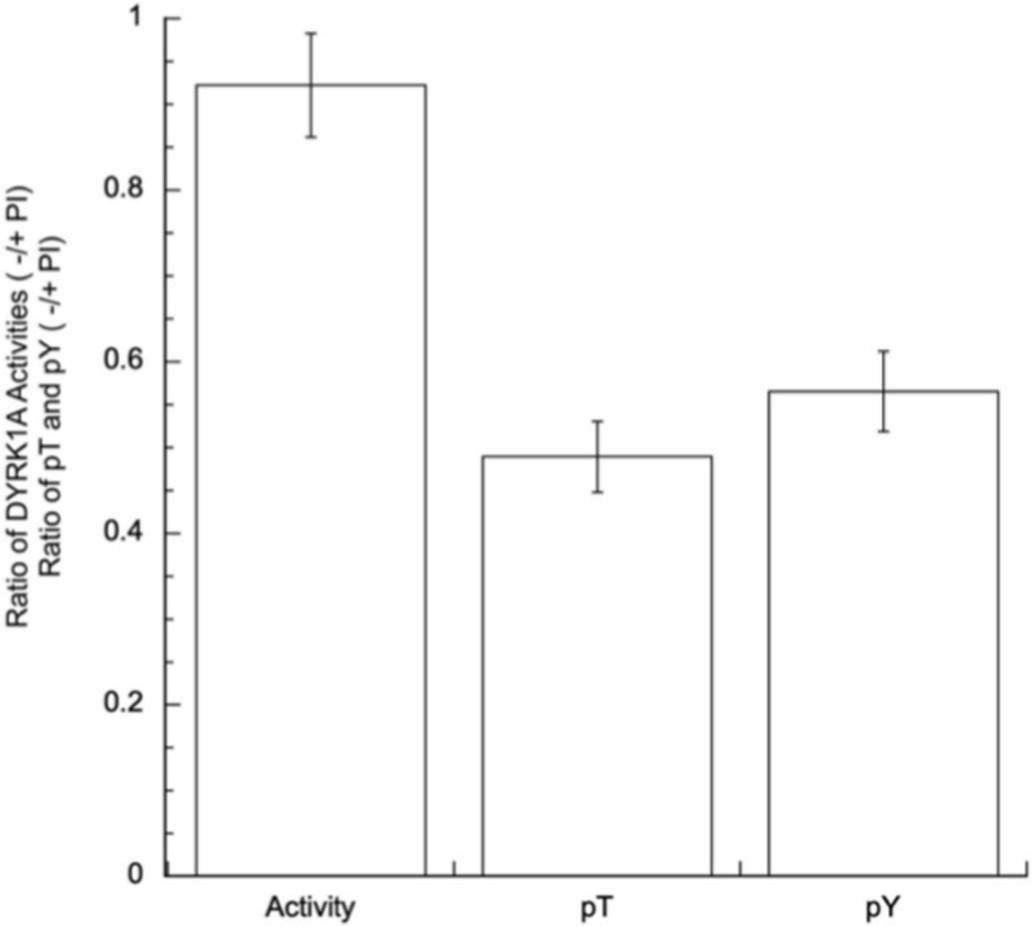
The effects of PI on phosphorylating activities and levels of phospho-amino acids in Ni-NTA eluents. Ni-NTA eluents were prepared either with or without PI from two cell lines GM07041 and GM07048. The resulting eluents were then subjected to DYRK1A assay and the phospho-amino acid measurement by probing with anti-phospho-amino acid antibodies in ELISA as described in Materials and Methods. The ratio of phosphorylating activity and phospho-amino acids between with and without PI were calculated from experiments performed in parallel on the same plate. Data presented were the mean ± SEM of the numbers of assays indicated. Ratio in DYRK1A activity (n = 8), in pT (n = 8) and in pY (n = 8).

We subsequently examined whether DYRK1A activity was affected by the state of phosphorylation in lysates. Crude lysates were prepared from normal cell lines with or without phosphatase inhibitors (PIs) in parallel. Ni-NTA eluents thus obtained were then analyzed for DYRK1A activity as described above to calculate the activity changed without PIs. As shown in Fig. 2, the DYRK1A activity was only marginally affected by omission of PIs during lysate preparation. In contrast, the same Ni-NTA eluents contains far less pT and pY when PIs are not present. It should be noted that we used anti-phospho-amino acid antibodies in combination with ELISA for measuring the levels of pY and pT, our data only reflects the general reduction in the levels of PY and pT, more specifically pT precedes a proline, not the phosphorylation at any specific location.

**Fig. 3:**
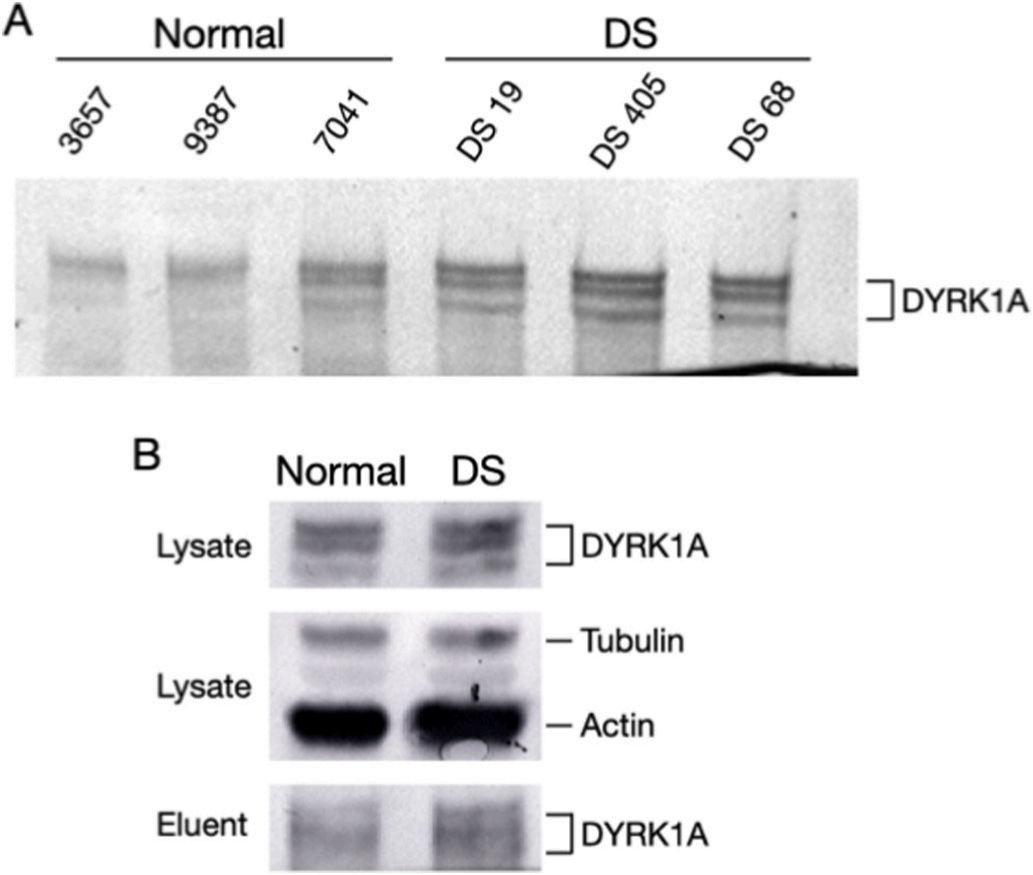
DYRK1A levels probed with Western blotting. A. DYRK1A levels in DS and normal cell lines. Lysate was prepared from cell lines as indicated and the same amount of each lysate (20 μg total protein) was subjected to Western blotting against antibody 8D9 as described in Materials and Methods. B. DYRK1A levels in samples before and after Ni-NTA purification. Cell lysates and Ni-NTA eluents prepared from DS and normal cell lines were probed for DYRK1A by Western. Tubulin and actin were used as loading controls for crude lysates.

**Fig. 4:**
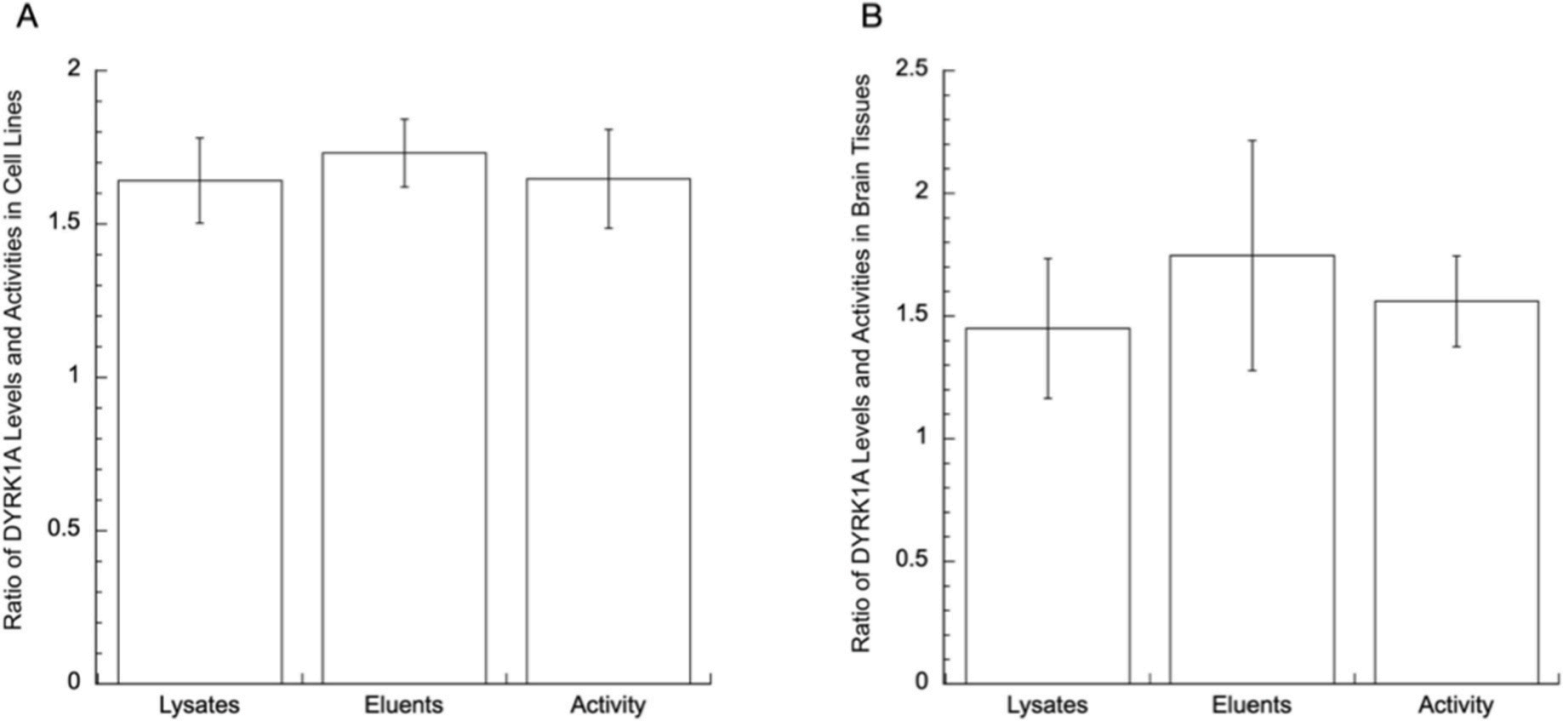
DYRK1A levels and activities in DS and normal subjects. A. Ratio of DYRK1A levels and activities between DS and normal cell lines. DYRK1A levels in crude lysates (Lysates) and Ni-NTA eluents (Eluents) were estimated semi-quantitatively by Western and used for calculating the ratio of DYRK1A levels between DS and normal. Only samples processed on the same blot were compared. For DRYK1A activity (Activity), phosphorylating activity in DS and normal Ni-NTA eluents were determined as described and used for calculating the ratio between DS and normal cells from experiments performed in parallel on the same plate. Data (Lysates, Eluents, and Activity) represents the mean ± SEM of 10 DS to normal measurements. B. Ratio of DYRK1A levels and activities between DS and normal brain tissues*.* Ni-NTA eluents were prepared from DS and normal brain tissues and analyzed as described in Fig. 4A. Data presented were the mean ± SEM of 4 DS to normal measurements.

*DYRK1A Activity in Control and DS.* We did a side-by-side comparison of protein level and activity for DYRK1A in control and DS samples to determine whether the difference in kinase activity deviates from that in protein level. Lysates were prepared from harvested cells, quantified, and then subjected to either Western blotting directly or to Ni-NTA purification for subsequent Western blotting and activity measurement as described. In agreement with our earlier findings, the level of DYRK1A in both crude lysates (Fig. 3A) and Ni-NTA eluents (Fig. 3B) was about 1.5x higher in DS versus control samples (Fig. 4A), reflecting the gene dosage-dependent difference in trisomy. Along with the level of protein, activity was also found to increase by the same ratio (Fig. 4A). Multiple normal and DS cell lines were employed for analysis, data presented are the average of results from the indicated numbers of random pairing of control and DS cells.

We also conducted side-by-side comparison of DYRK1A level and activity in brain tissue from control and DS subjects following the same approach as above. As shown in Fig. 4B, an approximately 1.5-fold increase in DYRK1A level was observed in DS versus control tissue. A similar fold increase was also observed in DYRK1A activity.

## 4 Discussion

We have developed an *in vitro* ELISA-based assay for measuring DYRK1A activity in cells and tissues. The assay utilizes the inherent property of DYRK1A containing a histidine stretch in its sequence for rapid enrichment of kinase from crude lysate using Ni-NTA resins. Ni-NTA eluents are then used to catalyze phosphorylation reaction in an established DYRK1A ELISA assay [22] for quantifying DYRK1A activity. Without Ni-NTA enrichment, background non-specific phosphorylation of the substrate proline-rich domain of dynamin 1, is too high in crude lysate to produce meaningful reading from the ELISA assay (data not shown). To further minimize the contribution of background activity, we incorporated a selective DYRK1A inhibitor, GCG, with a concentration able to nearly completely inhibit DYRK1A activity in the assay. We treat the readout that is suppressed by the inhibitor as the true DYRK1A activity. About 20% of the total phosphorylating activity in the Ni-NTA eluents is not susceptible to GCG, and so is considered to be non-specific. The finding was consistent as similar levels were observed across several cell lines and samples prepared at different days.

DYRK1A is elevated in DS in a gene-dosage dependent manner [4,5]. However, it is not known how changes in the level of DYRK1A and other cellular factors in DS are affecting kinase activity. This question was addressed in the current study. We measured the DYRK1A activity alongside DYRK1A levels in both control and DS subjects. The change in the in the activity of DYRK1A matches the level of DYRK1A (about 1.5-fold). No apparent difference was detected between them. The same result was observed in both cell lines (Fig. 4A) as well as in brain tissue (Fig. 4B).

We have conducted lysate preparation and Ni-NTA enrichment under native conditions. Various DYRK1A-associated proteins or cellular components, such as membrane vesicles [27] would likely be co-enriched with DYRK1A regardless of their role. This implies that DYRK1A in Ni-NTA eluents would be in similar conditions as in the cellular environments. Therefore, the parallel between the ratio of DYRK1A activity and protein levels in DS suggests that protein level is the main mechanism for regulation of DYRK1A activity. The one-pass Ni-NTA enrichment scheme is apparently not pulling down all cellular DYRK1A. Nevertheless, the ∼1.5-fold elevation of protein level in DS remains essentially the same before and after Ni-NTA enrichment suggests that the level of unbound DYRK1A (Ni-NTA FT in Fig. 1) would not alter our conclusion.

DYRK1A contains a^319^YQY^321^ motif in the activation segment [9,23], a structural equivalent of the TXY motif of MAPKs [9,28]. Phosphorylation of the threonine and tyrosine in the motif activates MAPK [29]. Tyrosine Y321 of the YQY motif is phosphorylated in mature DYK1A, regardless of the origins [9,30,31]. By analogy with the study of DYRK1A homologues, Drosophila DYRK2 and MNB, pY321 of DYRY1A is likely to be acquired through a one-off autophosphorylation mechanism during intermediate steps of translation before the proteins have properly folded [32]. However, the presence of pY321 is not required for DYRK1A catalytic activity as it can be fully removed in bacterially expressed recombinant DYRK1A without affecting kinase activity [31]. Here we show that DYRK1A in Ni-NTA eluents prepared from mammalian cells without PIs possesses similar activity as those with inhibitors, even though the levels of pY and pT of the preparation was reduced (Fig. 2). As we have only used phospho-amino acid antibodies for probing in this study, we do not know for certain which specific phospho-amino acids, including pY321, are removed. Nevertheless, our observations are consistent with the *in vitro* findings that pY as well as pT, whether on the activation loop or elsewhere on DYRK1A, are likely not directly involved in DYRK1A catalytic activity.

## Statements

All procedures were performed in compliance with relevant laws and institutional guidelines and have been approved by the appropriate institutional committees. Brain tissues used in this study were obtained from the brain bank of NYS Institute for Basic Research. The specimens are coded and the private information of donors is unidentifiable. Our experimental protocol was reviewed and approved (approval # 349) by IRB of the NYS Institute for Basic Research. The privacy rights of human subjects have been observed and that informed consent was obtained for experimentation with human subjects whenever applicable. This article did not use generative AI or AI-assisted technologies in writing.

## CRediT authorship contribution statement

**Wieslaw K. Dowjat:** Conceptualization, Data curation, Formal analysis, Writing – original draft, Writing – review & editing. **Yu-Wen Hwang:** Conceptualization, Data curation, Investigation, Writing – review & editing.

## Declaration of competing interest

The authors declare that they have no known competing financial interests or personal relationships that could have appeared to influence the work reported in this paper.

## Data Availability

Data will be made available on request.

## References

[1] Tejedor F., Zhu X.R., Kaltenbach E., Ackermann A., Baumann A., Canal I., Heisenberg M., Fischbach K.F., Pongs O. (1995). Minibrain: a new protein kinase family involved in postembryonic neurogenesis in *Drosophila*. Neuron.

[2] Guimerá J., Casas C., Pucharcòs C., Solans A., Domènech A., Planas A.M., Ashley J., Lovett M., Estivill X., Pritchard M.A. (1996). A human homologue of *Drosophila* minibrain (MNB) is expressed in the neuronal regions affected in Down syndrome and maps to the critical region. Hum. Mol. Genet..

[3] Antonarakis S.E., Skotko B.G., Rafii M.S., Strydom A., Pape S.E., Bianchi D.W., Sherman S.L., Reeves R.H. (2020). Down syndrome. Nat. Rev. Dis. Primers.

[4] Dowjat W.K., Adayev T., Kuchna I., Nowicki K., Palminiello S., Hwang Y.W., Wegiel J. (2007). Trisomy-driven overexpression of DYRK1A kinase in the brain of subjects with Down syndrome. Neurosci. Lett..

[5] Ait Yahya-Graison E., Aubert J., Dauphinot L., Rivals I., Prieur M., Golfier G., Rossier J., Personnaz L., Creau N., Blehaut H., Robin S., Delabar J.M., Potier M.C. (2007). Classification of human chromosome 21 gene-expression variations in Down syndrome: impact on disease phenotypes. Am. J. Hum. Genet..

[6] Dowjat K., Adayev T., Kaczmarski W., Wegiel J., Hwang Y.W. (2012). Gene dosage-dependent association of DYRK1A with the cytoskeleton in the brain and lymphocytes of Down syndrome patients. J. Neuropathol. Exp. Neurol..

[7] Murphy A.J., Wilton S.D., Aung-Htut M.T., McIntosh C.S. (2024). Down syndrome and DYRK1A overexpression: relationships and future therapeutic directions. Front. Mol. Neurosci..

[8] Himpel S., Tegge W., Frank R., Leder S., Joost H.G., Becker W. (2000). Specificity determinants of substrate recognition by the protein kinase DYRK1A. J. Biol. Chem..

[9] Soundararajan M., Roos A.K., Savitsky P., Filippakopoulos P., Kettenbach A.N., Olsen J.V., Gerber S.A., Eswaran J., Knapp S., Elkins J.M. (2013). Structures of Down syndrome kinases, DYRKs, reveal mechanisms of kinase activation and substrate recognition. Structure.

[10] Arbones M.L., Thomazeau A., Nakano-Kobayashi A., Hagiwara M., Delabar J.M. (2019). DYRK1A and cognition: a lifelong relationship. Pharmacol. Ther..

[11] Woods Y.L., Cohen P., Becker W., Jakes R., Goedert M., Wang X., Proud C.G. (2001). The kinase DYRK phosphorylates protein-synthesis initiation factor eIF2Bepsilon at Ser539 and the microtubule-associated protein tau at Thr212: potential role for DYRK as a glycogen synthase kinase 3-priming kinase. Biochem. J..

[12] Ryoo S.R., Jeong H.K., Radnaabazar C., Yoo J.J., Cho H.J., Lee H.W., Kim I.S., Cheon Y.H., Ahn Y.S., Chung S.H., Song W.J. (2007). DYRK1A-mediated hyperphosphorylation of Tau. A functional link between Down syndrome and alzheimer disease. J. Biol. Chem..

[13] Wegiel J., Gong C.X., Hwang Y.W. (2011). The role of DYRK1A in neurodegenerative diseases. FEBS J..

[14] Ryoo S.R., Cho H.J., Lee H.W., Jeong H.K., Radnaabazar C., Kim Y.S., Kim M.J., Son M.Y., Seo H., Chung S.H., Song W.J. (2008). Dual-specificity tyrosine(Y)-phosphorylation regulated kinase 1A-mediated phosphorylation of amyloid precursor protein: evidence for a functional link between Down syndrome and alzheimer's disease. J. Neurochem..

[15] Aslam A.A., Baksh R.A., Pape S.E., Strydom A., Gulliford M.C., Chan L.F., Consortium G.-D. (2022). Diabetes and obesity in Down syndrome across the lifespan: a retrospective cohort study using U.K. electronic health records. Diabetes Care.

[16] Heit J.J., Apelqvist A.A., Gu X., Winslow M.M., Neilson J.R., Crabtree G.R., Kim S.K. (2006). Calcineurin/NFAT signalling regulates pancreatic beta-cell growth and function. Nature.

[17] Gwack Y., Sharma S., Nardone J., Tanasa B., Iuga A., Srikanth S., Okamura H., Bolton D., Feske S., Hogan P.G., Rao A. (2006). A genome-wide drosophila RNAi screen identifies DYRK-family kinases as regulators of NFAT. Nature.

[18] Wang P., Alvarez-Perez J.C., Felsenfeld D.P., Liu H., Sivendran S., Bender A., Kumar A., Sanchez R., Scott D.K., Garcia-Ocana A., Stewart A.F. (2015). A high-throughput chemical screen reveals that harmine-mediated inhibition of DYRK1A increases human pancreatic beta cell replication. Nat. Med..

[19] Kida E., Walus M., Jarzabek K., Palminiello S., Albertini G., Rabe A., Hwang Y.W., Golabek A.A. (2011). Form of dual-specificity tyrosine-(Y)-phosphorylation-regulated kinase 1A nonphosphorylated at tyrosine 145 and 147 is enriched in the nuclei of astroglial cells, adult hippocampal progenitors, and some cholinergic axon terminals. Neuroscience.

[20] Huang Y., Chen-Hwang M.C., Dolios G., Murakami N., Padovan J., Wang R., Hwang Y.W. (2004). Mnbk/Dyrk1A phosphorylation regulates the interaction of dynamin 1with SH3 domain-containing proteins. Biochemistry.

[21] Tosato G., Cohen J.I. (2007). Generation of Epstein-Barr virus (EBV)-immortalized B cell lines. Curr. Protoc. Immunol. Chapt..

[22] Liu Y., Adayev T., Hwang Y.W. (2017). An ELISA DYRK1A non-radioactive kinase assay suitable for the characterization of inhibitors. F1000Res.

[23] Kentrup H., Becker W., Heukelbach J., Wilmes A., Schürmann A., Huppertz C., Kainulainen H., Joost H.G. (1996). Dyrk, a dual specificity protein kinase with unique structural features whose activity is dependent on tyrosine residues between subdomains VII and VIII. J. Biol. Chem..

[24] Wang J., Kudoh J., Shintani A., Minoshima S., Shimizu N. (1998). Identification of two novel 5' noncoding exons in human MNB/DYRK gene and alternatively spliced transcripts. Biochem. Biophys. Res. Commun..

[25] Guimerá J., Casas C., Estivill X., Pritchard M. (1999). Human minibrain homologue (MNBH/DYRK1): characterization, alternative splicing, differential tissue expression, and overexpression in Down syndrome. Genomics.

[26] Araldi G.L., Hwang Y.W. (2022). Design, synthesis, and biological evaluation of polyphenol derivatives as DYRK1A inhibitors. The discovery of a potentially promising treatment for multiple sclerosis. Bioorg. Med. Chem. Lett.

[27] Murakami N., Bolton D., Hwang Y.W. (2009). Dyrk1A binds to multiple endocytic proteins required for formation of clathrin-coated vesicles. Biochemistry.

[28] Canagarajah B.J., Khokhlatchev A., Cobb M.H., Goldsmith E.J. (1997). Activation mechanism of the MAP kinase ERK2 by dual phosphorylation. Cell.

[29] Payne D.M., Rossomando A.J., Martino P., Erickson A.K., Her J.H., Shabanowitz J., Hunt D.F., Weber M.J., Sturgill T.W. (1991). Identification of the regulatory phosphorylation sites in pp42/mitogen-activated protein kinase (MAP kinase). EMBO J..

[30] Himpel S., Panzer P., Eirmbter K., Czajkowska H., Sayed M., Packman L.C., Blundell T., Kentrup H., Grotzinger J., Joost H.G., Becker W. (2001). Identification of the autophosphorylation sites and characterization of their effects in the protein kinase DYRK1A. Biochem. J..

[31] Adayev T., Chen-Hwang M.C., Murakami N., Lee E., Bolton D.C., Hwang Y.W. (2007). Dual-specificity tyrosine phosphorylation-regulated kinase 1A does not require tyrosine phosphorylation for activity in vitro. Biochemistry.

[32] Lochhead P.A., Sibbet G., Morrice N., Cleghon V. (2005). Activation-loop autophosphorylation is mediated by a novel transitional intermediate form of DYRKs. Cell.

